# Advances in *MICA* genotyping: characterization of 406 novel alleles and their frequencies in multiple populations

**DOI:** 10.3389/fimmu.2026.1741611

**Published:** 2026-03-02

**Authors:** Viviane Albrecht, Christin Paech, Kathrin Putke, Gerhard Schöfl, Jürgen Sauter, Alexander H. Schmidt, Vinzenz Lange, Anja Klussmeier

**Affiliations:** 1 DKMS Life Science Lab, Dresden, Germany; 2 DKMS Group, Tübingen, Germany

**Keywords:** allele, genotyping, HLA, *MICA*, population frequency

## Abstract

In 2020, we reported *MICA* allele frequencies from a cohort of over one million German individuals. This study identified MICA*008 (42%), MICA*002 (12%), and MICA*009 (9%) as the most common *MICA* alleles at protein resolution. Additionally, we discovered novel alleles with a cumulative frequency of 0.3%. To reduce this fraction of unnamed sequences, we aimed to fully characterize the most frequent novel alleles using both long- and short-read sequencing. As a result, we submitted 603 sequences to the IPD-IMGT/HLA Database: 406 novel alleles and 197 sequence extensions and confirmations. Among the novel alleles, 199 encoded for distinct novel MICA proteins. Following the inclusion of these sequences into the IPD-IMGT/HLA Database, we genotyped 93,814 individuals from an independent cohort. In the German subset (n=48,618), our previous findings on *MICA* allele frequencies were confirmed. As anticipated, the cumulative frequency of novel alleles decreased significantly from 0.3% to 0.03%, reflecting the expanded reference database. The most frequent of the previously novel alleles were MICA*107N (0.02%), MICA*141 (0.01%), MICA*119 (0.01%), MICA*089 (0.01%), and MICA*247 (0.01%). While allele frequencies in other European and the South African White population were similar to those in Germany, greater variation was observed in the South African Black, non-indigenous Chilean, and Turkish populations. Notably, some of the novel alleles appeared to be population-specific; for example, MICA*258 was exclusively identified in samples from the Black or Colored populations of South Africa. In conclusion, the extensive characterization of novel *MICA* alleles has substantially reduced the fraction of unknown sequences in *MICA* donor genotyping, which will support future biomedical and population genetic studies.

## 1 Introduction

The *MICA* (MHC class I polypeptide-related sequence A) gene is located on chromosome 6 within the human major histocompatibility (MHC) complex, between *HLA-B* and *MICB* (1). Although structurally similar to the classical human leukocyte antigen (HLA) genes, MICA does not present peptides. Upon stress, various cell types (e.g., epithelial cells, fibroblasts) upregulate expression of *MICA*, which activates the NKG2D receptor on NK cells and T cell subsets. Consequently, MICA promotes immune cell recognition and immune surveillance (2–4). However, MICA can also be shed from the cellular surface as soluble MICA (sMICA), thereby decreasing NKG2D activation (5, 6).

The *MICA* gene is encoded by six exons. Exon 1 encodes the leader peptide, exons 2–4 the extracellular domain, exon 5 the transmembrane domain and exon 6 the cytoplasmatic tail (1, 7). Like classical HLA genes, *MICA* is polymorphic. Prior to this work, the IPD-IMGT/HLA Database described 107 *MICA* alleles (among them 84 distinct MICA proteins), of which only 15 (14%) were described in full length from 5’ to 3’ UTR (release 3.35, January 2019).

*MICA* alleles can be grouped based on polymorphisms that influence function. One major group consists of MICA*008-like alleles. MICA*008, the most frequent allele in many populations, has a frameshift mutation in exon 5, which leads to the loss of the transmembrane domain. Nevertheless, it is still attached to the cell surface via a GPI-anchor (8). After exosomal release, it has been reported to downregulate the NKG2D response more efficiently than the transmembrane-bound alleles, which are shed as sMICA by proteolytic cleavage (9, 10). In general, both types of sMICA decrease MICA cell surface expression and thereby NKG2D activation. This has been associated with inferior outcome in tumor patients and may represent a cancer immune evasion principle (5, 6). Another important polymorphism is the methionine/valine (Met/Val) dimorphism at position 129 of the mature MICA protein (rs1051792; MICA-129), which stratifies *MICA* alleles into high-affinity (Met) and low-affinity (Val) binders to NKG2D (11, 12). MICA-129 has been linked to susceptibility or protection in various autoimmune diseases, cancers, and viral infections (13, 14). In hematopoietic cell transplantation (HCT) and kidney transplantation, *MICA* allele matching or MICA-129 matching has been associated with a favorable outcome for the patient, e.g., a decrease in acute graft-versus host disease (GVHD) (15–20). Despite this data, current guidelines for HCT do not yet recommend *MICA*-informed donor selection (21, 22). Nonetheless, due to strong linkage disequilibrium between *MICA* and *HLA-B*, over 90% of 10/10 HLA-matched donor-recipient pairs are also matched at the *MICA* locus (17, 23).

To enable broader studies of *MICA* informed donor selection in unrelated allogenic HCT, we started to genotype potential stem cell donors for *MICA* upon registration in 2017. In 2020, we published *MICA* allele frequencies for the German population based on over one million samples. The five most frequent alleles were MICA*008 (42%), MICA*002 (12%), MICA*009# (9%), MICA*010# (8%) and MICA*004 (7%) (24). In that study, we identified novel *MICA* alleles with a cumulative allele frequency of 0.3%. As expected for a gene that had not yet been broadly genotyped, this value was about tenfold higher than the rate observed for classical HLA genes (e.g., 0.02% for HLA class I genes and 0.04% HLA class II genes (based on sequencing of exon 2 and 3 only); unpublished data). These unnamed alleles complicate genotyping and cannot be clinically reported, thereby limiting their utility in donor selection when *MICA* matching is relevant. To address this situation and simplify future *MICA* genotyping, we aimed to characterize the most frequent novel *MICA* alleles and submit them to the IPD-IMGT/HLA Database.

## 2 Methods

### 2.1 Samples

Volunteers from Germany, Poland, UK, USA, Chile, India and South Africa continuously provide samples (buccal swabs) to DKMS for their registration as potential stem cell donors. Between 2017 and 2021, approximately 3.6 million samples were genotyped for *MICA* (Germany 56%; Poland 18%; UK 15%; US 8%; Chile 2%; India 1%; South Africa 0% (donor center not yet active)). This cohort was used to identify and characterize novel alleles. Another 93,814 samples were genotyped for *MICA* from 2023 to 2024 and used for *MICA* population frequency analyses (Germany 65%; Poland 13%; South Africa 8%, Chile 7%; UK 4%; US 3%; India 0.2%). As part of the registration process, the donors are asked to self-assign their ethnic background. All subjects gave written informed consent in accordance with the Declaration of Helsinki. The described genotyping is within the scope of the consent forms signed at recruitment.

### 2.2 High-throughput genotyping

Samples for the registration of potential stem cell donors are genotyped in an high-throughput workflow that targets *HLA-A*, *-B*, *-C*, *-E*, *-DPB1*, *-DQB1*, *-DRB1*, *-DPA1*, *-DQA1*, *-DRB3/4/5*, *MICA* and *MICB* (MICA/B), KIR, blood groups ABO and Rh, and *CCR5* as described before (24–30). A detailed description of the workflow with a focus on *MICA* genotyping can be found in Klussmeier et al. (24). In brief, *MICA* exons 2, 3, 4, 5 are amplified by PCR (complete coverage of exons 2 and 3, partial coverage of exons 4 and 5). After pooling the PCR products with the HLA loci of the same donor, an indexing PCR is performed. Before 2019, the PCR products of up to 3,840 donors were pooled, cleaned up and sequenced using HiSeq Rapid SBS Kits V2 (500 cycles) on HiSeq2500 instruments (Illumina, San Diego, USA). After 2019, up to 7520 potential stem cell donors were sequenced using NovaSeq6000 SP Kits (500 cycles) on a NovaSeq6000 instrument (Illumina, San Diego, USA). Genotyping of high-throughput sequencing data was performed by neXtype (24, 25). Since not all bases of *MICA* are covered by our workflow, some genotyping results are ambiguous. Here, we report them by a representative allele, which is marked with a hash symbol (#) (**Table 1**). Previously, we described haplotypes with *MICA* duplications and *MICA* deletions (31). While neXtype correctly genotypes *MICA* duplications and reports three *MICA* alleles in such samples, it reports a homozygous instead of a hemizygous result for samples with *MICA* deletions. Nevertheless, since *MICA* deletions are rare (e.g., 0.3% in Europe, 2.5% in Chile), we accepted that this might minimally influence allele frequency calculations.

**Table 1:** Overview of ambiguous genotyping results.

Allele group	Alleles
MICA*002#	MICA*002, MICA*110
MICA*009#	MICA*009, MICA*049
MICA*010#	MICA*010, MICA*065, MICA*069
MICA*027#	MICA*027, MICA*048
MICA*047#	MICA*047, MICA*101

### 2.3 Novel allele characterization and submission

Samples with novel *MICA* alleles were subjected to two independent long-range PCRs (12 kB) that amplify the complete *MICA* gene from 5’ to 3’UTR. The following primers were used: CTGCTTGAGCCGCTGAGAGG (forward), GATCCAGGCAGGGAATTGAATCCC and GAGATCCAGGCAGGGAATTCAATTCC (reverse). In detail, 4 μL genomic DNA was combined with 0.08 μM primer mix, 1x Advantage Genomic LA Buffer, 1.25 U Advantage Genomic LA Polymerase Mix (Takara Bio, Mountain View, California), and dNTPs (0.4 mM each) in a 25 μL reaction volume. PCR conditions: 94 °C 1 minute, 35 cycles: 98 °C 10 seconds/65 °C 12 minutes, 72 °C 10 minutes. PCR success was checked by agarose gel electrophoresis. The product of one PCR reaction was used for Illumina shotgun sequencing as described before (32–34). In brief, fragmentation and adapter ligation was performed according to “NEBNext Ultra II DNA Library Prep Kit for Illumina” protocol (New England Biolabs, Ipswich, Massachusetts). After purification with 0.7x SPRIselect beads (Beckman Coulter, Brea, California), custom barcodes were attached by a 7-cycle-indexing PCR. Finally, 48 samples were pooled and subsequently purified using 0.7x SPRIselect beads. After qPCR library quantification, four pools (up to 192 samples) were sequenced on a MiSeq instrument using a MiSeq Reagent Kit v2 (500 cycles) according to the manufacturer’s instructions (Illumina, San Diego, California). The product of the second PCR reaction was used for SMRT sequencing (Pacific Biosciences, Menlo Park, California) as described before (32). PCR products of the prior long-range PCR were barcoded by an additional 10-cycle PCR reaction with indexing primers (0.2 μM). 192 samples were then pooled and library preparation was carried out according to the manufacturer’s instructions. Libraries were size selected with the BluePippin system using a 0.75% cartridge (Sage Science, Beverly, Massachusetts) and sequenced on a Sequel instrument using Sequel Sequencing Kit 3.0, SMRT Cell 1 M v3 and a 10 hour movie (Pacific Biosciences, Menlo Park, California).

Sequencing reads were analyzed using NGSengine (GenDx, Utrecht, The Netherlands) and dual redundant reference sequencing (DR2S) as described before (35, 36). The versions of the IPD-IMGT/HLA Database used in this analysis ranged from release 3.35 (2019) to release 3.48 (2022), with each sample batch analyzed using the most current version available at the time (refer to the analysis date of individual sequences in **Supplementary Data**). Samples with low sequencing quality and not fully conserved consensus sequences were discarded from analysis. Finally, all approved sequences were submitted to the IPD-IMGT/HLA Database using TypeLoader2 (37, 38). In general, all novel sequences were submitted. In addition, we submitted sequence extensions for alleles so far only partially described in the IPD-IMGT/HLA Database. Often, either was true for both alleles of a sequenced sample. If two identical sequences from different samples were available, the second sequence was submitted as confirmation.

In general, samples that failed in PCR and/or analysis were not repeated. We know from experience that this is usually caused by insufficient DNA quality, especially DNA fragmentation, and will not improve by repetition. To deal with this issue, three to five samples with the same targeted novel variation were selected for sequencing if enough samples were available.

### 2.4 Alignment

MICA protein sequences were obtained from the IPD-IMGT/HLA Database (release 3.60) and aligned using CLC Genomics Workbench (version 24.0) (Qiagen Digital Insights, Aarhus, Denmark). Only sequences with complete amino acid coverage were included. A custom R script was used to compare every amino acid in the alignment to the corresponding amino acid of the reference allele MICA*002. Finally, alleles were sorted manually to generate clusters that visually highlight the similarity of alleles to the most frequent ones.

### 2.5 Phylogenetic tree

A distance matrix was calculated from the alignment using hamming distance and a neighbor-joining tree with midpoint rooting was built using the R package ape version 5.8.1 (39). Sequences without complete amino acid coverage and null alleles were excluded. Visualization was performed using the R package ggtree version 3.14.0 (40). For improved visualization, the branch lengths of the tree were square rooted before plotting the tree.

### 2.6 Frequency calculations

*MICA* population frequencies were calculated using samples that were genotyped in the high-throughput workflow with IPD-IMGT/HLA Database release 3.50 or higher. At this time (January 2023), all our submitted novel exon variations were officially named by the IPD-IMGT/HLA Database and consequently used for genotyping by neXtype. As part of the registration process as potential stem cell donors, the donors are asked to self-assign their ethnic background. These data were used for calculating *MICA* population frequencies. Since selectable ethnicities varied between the different DKMS donor center questionnaires, data were only grouped within one donor center (e.g., samples indicated as DE_Turkey were collected in Germany but the donor self-assigned to a Turkish ethnic background). Populations with more than 1,000 genotyped samples were selected for calculating *MICA* frequencies (DE_Germany, PL_Poland, ZA_Black, CL_Non-Indigenous, UK_British/Irish, ZA_White, DE_Turkey). Due to lacking sequence information outside of exons 2-5, *MICA* population frequencies were only calculated at protein resolution (first field). For samples with phasing ambiguities, the probability of each possible result was calculated based on the allele frequencies of unambiguously typed samples in the respective population. According to these probabilities, counts were added to the different alleles. Ambiguities that cannot be resolved by our workflow are listed in **Table 1**.

## 3 Results

### 3.1 *MICA* sequencing and submission

**Figure 1:**
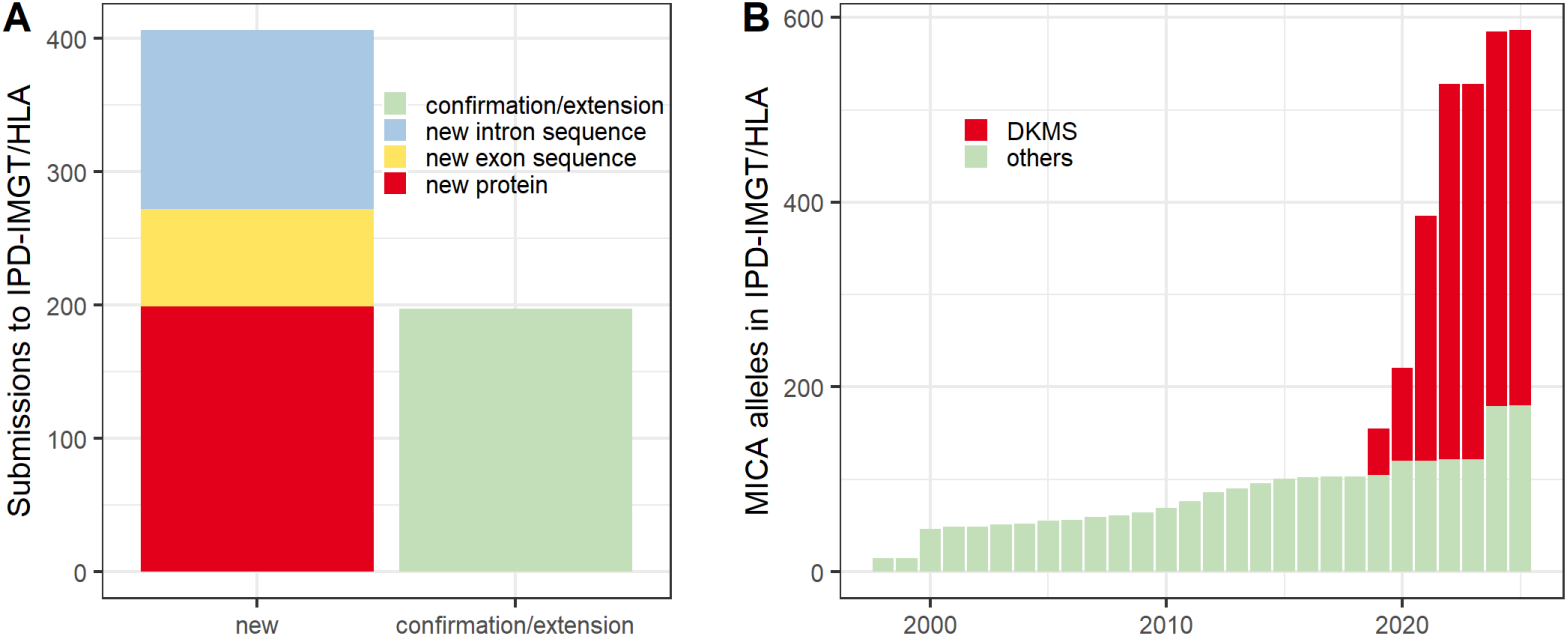
Submission to the IPD-IMGT/HLA Database. **(A)** Number of *MICA* sequences submitted to the IPD-IMGT/HLA Database. **(B)** Number of *MICA* alleles in the IPD-IMGT/HLA Database over time.

In 2017, we added *MICA* genotyping to our high-throughput stem cell donor workflow. At that time, 107 *MICA* alleles were listed in the IPD-IMGT/HLA Database, of which 92 (86%) were only partially described (release 3.35, January 2019). Because partial allele entries in the database can complicate genotyping, our initial goal was to extend the sequences of frequently observed partial *MICA* alleles in the IPD-IMGT/HLA Database. Hence, we selected 299 samples with partial sequence coverage and sequenced *MICA* in full-length. Thereby, each targeted allele was covered by multiple samples. After sequence analysis, we could successfully extend the sequences of 35 distinct, previously only partially described, *MICA* alleles. Overall, this first batch resulted in 209 sequence submissions to the IPD-IMGT/HLA Database, among them 22 alleles coding for novel MICA proteins, 9 synonymous exon variations, 70 intron variations and 108 confirmations/sequence extensions (**Supplementary Data**). These alleles were incorporated in the IPD-IMGT/HLA Database releases between January and October 2020. By release 3.42, the number of *MICA* alleles had increased to 224, of which 159 were described in full-length (71%) (**Figure 1**).

By 2020, we had genotyped approximately 3.6 million samples, of which 11,091 contained a novel sequence (0.3%) in exons 2, 3, 4, or 5. However, some of these novel sequences were observed repeatedly, e.g. the most frequent novel sequences were identified in 1,273 and 763 samples (these sequences were later named MICA*141 and MICA*119, respectively). Overall, we identified 1,103 distinct novel sequences of which 145 were detected more than ten times. In contrast, 559 variations were observed only once and are presumably very rare alleles.

For optimal use of our resources, we focused the second batch of novel *MICA* allele characterization on the 145 most frequent variants. A total of 474 samples were selected to cover each variation with multiple samples. Lower-frequency variations were added only to fill plates. As expected from prior experience of long-range PCRs on buccal swab derived DNA, approximately 33% of samples failed (25% in PCR, 8% in analysis) (34), likely due to DNA fragmentation. However, reasons for PCR failure were not further investigated for individual samples. Following analysis, this second batch of *MICA* novel allele characterization led to 394 sequence submissions to the IPD-IMGT/HLA Database, among them 177 alleles coding for novel proteins, 64 synonymous exon variations, 64 intron variations and 89 confirmations/sequence extensions. These include 139 (96%) of the targeted 145 frequent variations.

Combining both batches, we submitted 603 sequences to the IPD-IMGT/HLA Database: 199 novel proteins, 73 synonymous exon variations, 134 intron variations, and 197 confirmations/sequence extensions (**Figure 1A**). These sequences now represent approximately two-thirds of all *MICA* alleles listed in the IPD-IMGT/HLA Database (release 3.60, April 2025) (**Figure 1B**).

### 3.2 Novel *MICA* proteins

Among the characterized and submitted *MICA* alleles were 199 coding for novel MICA proteins. A detailed overview of all base variations in comparison to the closest known allele at the time of sequence submission is provided in **Supplementary Data**.

**Figure 2:**
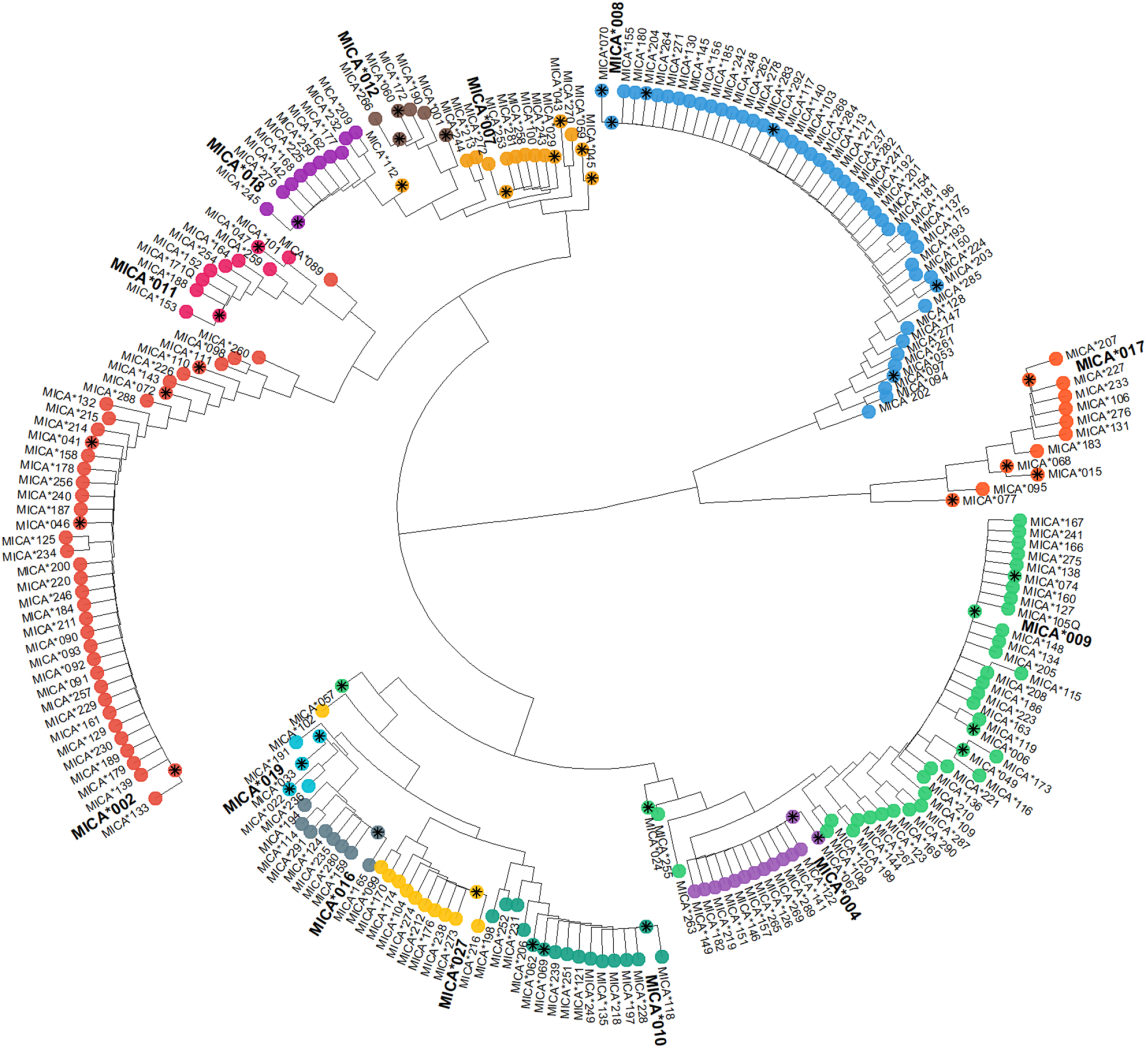
Phylogenetic tree. All MICA amino acid sequences with available full-length coverage (IPD-IMGT/HLA release 3.60) are displayed as neighbor-joining tree with midpoint rooting. The 13 most frequent MICA alleles in Germany are highlighted in large and bold font. Colored tips depict groups of similar alleles. Asterisks in the tip of the alleles indicate that the allele was either previously present in the IPD-IMGT/HLA Database or has been submitted by another laboratory. All alleles without the asterisk resulted from the present work. Null alleles are not shown.

**Figure 3:**
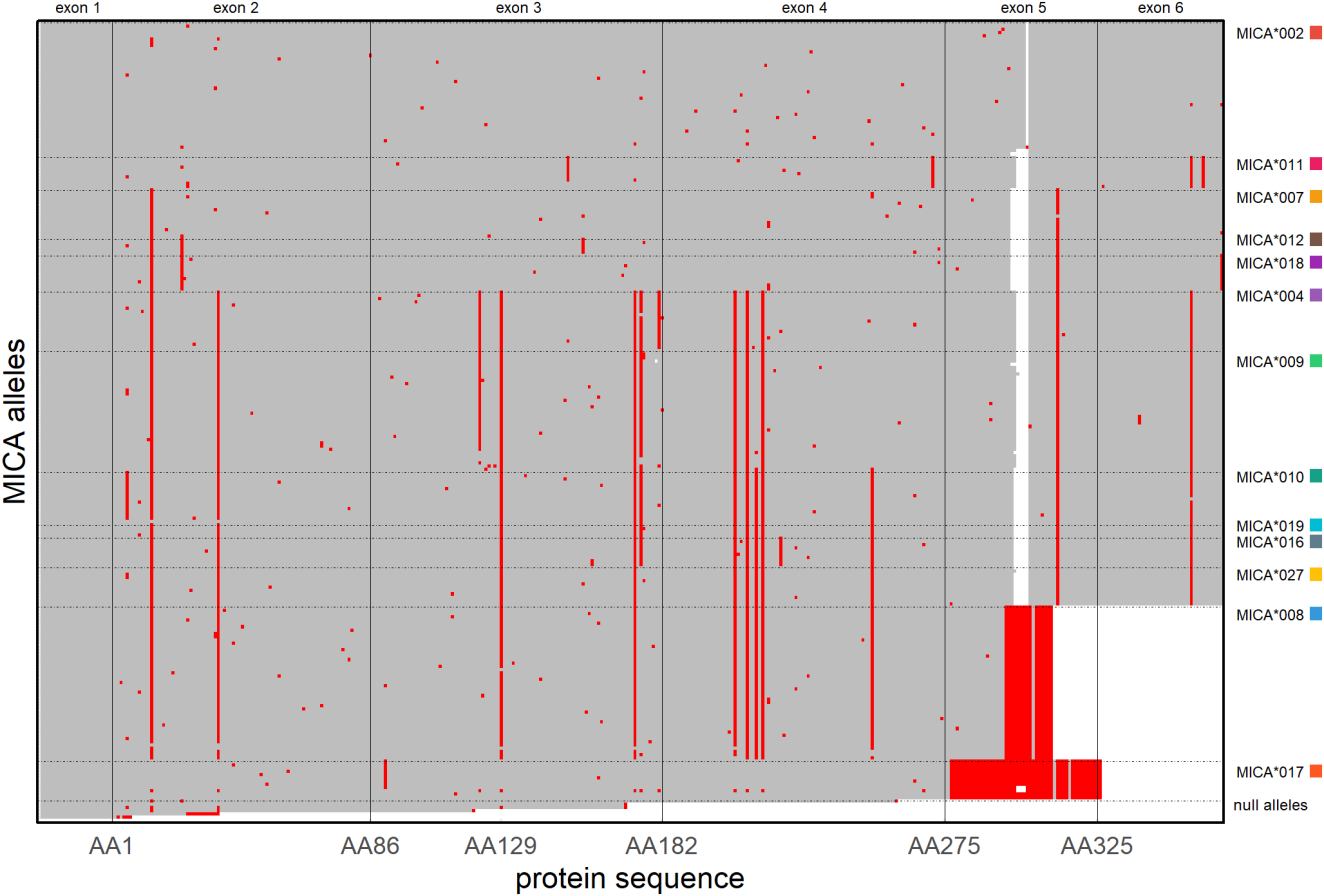
Alignment of MICA proteins. All 230 MICA full-length protein sequences (IPD-IMGT/HLA Database release 3.60) were aligned. Amino acids identical to the reference allele MICA*002 are depicted in grey, differing amino acids are depicted in red, deletions in white. MICA proteins were grouped by recurrent variations (dashed horizontal lines) with the most frequent member of a group given on the right (colored squares correspond to colors used in **Figure 2**). Exon boundaries are indicated by vertical lines. Amino acids are numbered by the mature MICA protein with amino acid 1 (AA1) being encoded by the first codon of exon 2. The Met/Val dimorphism is located at position AA129. A larger version of the figure with individual allele annotations is added as **Supplementary Figure 1**.

At first-field (protein) resolution, MICA*008, MICA*002 and MICA*009 have been identified as the most common alleles in the German population (24). Consequently, it is not surprising that more than half of the submitted novel alleles are variations of these alleles (**Figures 2**, **3**). Nevertheless, we identified variations of all other frequent *MICA* alleles. Specifically, most of the previously undescribed amino acid variations appear to be randomly distributed within the regions covered by our high throughput genotyping workflow (amino acids 1–181 and 204–319 of the mature protein) (**Figure 3**).

The most extensively studied amino acid variation in MICA is the Met/Val dimorphism at position 129. Among the common alleles, MICA*002, MICA*007, MICA*011, MICA*012, MICA*017, and MICA*018 encode a methionine at this position, while MICA*004, MICA*008, MICA*009, MICA*010, MICA*016, MICA*019, and MICA*027 encode valine (**Figure 3**). Notably, two of our novel alleles are exceptions regarding this amino acid. While MICA*147 and MICA*202 are otherwise very similar to the valine-encoding MICA*008 (**Figure 2**), they encode methionine at position 129.

Additionally, we report five new *MICA* null alleles. Frameshift mutations in MICA*096N and MICA*107N are present in exon 2, while those of MICA*195N, MICA*222N, and MICA*286N are located in exon 3 (**Figure 3**; **Supplementary Data**). These mutations are predicted to result in non-functional proteins.

### 3.3 *MICA* alleles in different populations

In 2020, we published *MICA* allele frequencies for the German population based on over one million samples, identifying novel alleles at a cumulative allele frequency of 0.3% (24). After characterization of the most frequent novel alleles, our next objective was to analyze the allele frequencies of the previously novel alleles across different populations.

Our independent new cohort consisted of 93,814 samples genotyped for *MICA* between 2023 and 2024 using our high-throughput workflow. Within this cohort, we identified seven populations with over 1000 samples each: DE_Germany (n=48,618), PL_Poland (n=11,776), CL_Non-Indigenous (n=4,937), ZA_Black (n=4,085), UK_British/Irish (n=2,090), ZA_White (n=1,989) and DE_Turkey (n=1,823). These samples were used to calculate allele frequencies at protein resolution (first field).

Our largest population, DE_Germany, confirmed the results from our previous study (**Table 2**) (24). The most frequent *MICA* allele was MICA*008 (44%), followed by MICA*002# (11%), MICA*009# (9%), MICA*010# (8%), and MICA*004 (7%). Among the previously novel *MICA* alleles, the most frequent alleles in the German population were MICA*107N (0.02%), MICA*141, MICA*089, MICA*119 and MICA*136 (all 0.01%) (**Table 2**; **Supplementary Data**).

**Table 2:** *MICA* allele frequencies across populations.

MICA Allele	Novel	frequency DE_Germany n=48618	frequency PL_Poland n=11776	PL/DE	frequency UK_British/Irish n=2090	UK/DE	frequency ZA_White n=1989	ZAW/DE	frequency ZA_Black n=4085	ZAB/DE	frequency CL_Non-Indigenous n=4937	CL/DE	frequency DE_Turkey n=1823	Turkey/DE
MICA*008	N	0.43643	0.39556	0.91	0.50505	1.16	0.43489	1.00	0.27694	0.63	0.19437	0.45	0.21419	0.49
MICA*002#	N	0.11496	0.13317	1.16	0.08822	0.77	0.12418	1.08	0.22484	1.96	0.31115	2.71	0.14243	1.24
MICA*009#	N	0.08733	0.09382	1.07	0.06250	0.72	0.07692	0.88	0.04536	0.52	0.08630	0.99	0.19687	2.25
MICA*010#	N	0.07777	0.05242	0.67	0.06370	0.82	0.07567	0.97	0.00049	0.01	0.06604	0.85	0.02337	0.30
MICA*004	N	0.06501	0.07199	1.11	0.07644	1.18	0.07466	1.15	0.24470	3.76	0.10432	1.60	0.08111	1.25
MICA*007	N	0.04845	0.06378	1.32	0.04736	0.98	0.03796	0.78	0.00221	0.05	0.01570	0.32	0.02475	0.51
MICA*018	N	0.03602	0.05950	1.65	0.02188	0.61	0.03042	0.84	0.02869	0.80	0.02330	0.65	0.07204	2.00
MICA*017	N	0.03321	0.03390	1.02	0.03413	1.03	0.03142	0.95	0.00025	0.01	0.01854	0.56	0.01320	0.40
MICA*012	N	0.02145	0.02338	1.09	0.02380	1.11	0.01961	0.91	0.01839	0.86	0.01276	0.59	0.04482	2.09
MICA*016	N	0.01886	0.02238	1.19	0.00697	0.37	0.02187	1.16	0.00196	0.10	0.02188	1.16	0.09926	5.26
MICA*011	N	0.01846	0.01115	0.60	0.02788	1.51	0.03117	1.69	0.01618	0.88	0.04487	2.43	0.02255	1.22
MICA*027#	N	0.01577	0.01856	1.18	0.00913	0.58	0.01232	0.78	0.00037	0.02	0.04659	2.96	0.02035	1.29
MICA*019	N	0.00838	0.00578	0.69	0.01875	2.24	0.00804	0.96	0.05431	6.48	0.01509	1.80	0.00605	0.72
MICA*001	N	0.00778	0.00352	0.45	0.00889	1.14	0.00930	1.20	0.01349	1.73	0.02036	2.62	0.00055	0.07
MICA*006	N	0.00425	0.00436	1.03	0.00120	0.28	0.00302	0.71	0.00000	0.00	0.00162	0.38	0.02942	6.92
MICA*015	N	0.00079	0.00063	0.80	0.00072	0.91	0.00251	3.19	0.04634	58.75	0.00436	5.52	0.00137	1.74
MICA*029	N	0.00069	0.00042	0.61	0.00000	0.00	0.00025	0.37	0.00025	0.36	0.00051	0.74	0.00110	1.60
MICA*068	N	0.00054	0.00063	1.16	0.00048	0.89	0.00000	0.00	0.00907	16.71	0.00334	6.16	0.00027	0.51
MICA*047#	N	0.00031	0.00063	2.05	0.00024	0.78	0.00025	0.82	0.00000	0.00	0.00091	2.97	0.00082	2.68
MICA*072	N	0.00031	0.00025	0.82	0.00024	0.78	0.00000	0.00	0.00000	0.00	0.00000	0.00	0.00000	0.00
MICA*045	N	0.00028	0.00008	0.30	0.00024	0.87	0.00126	4.54	0.00049	1.77	0.00051	1.83	0.00110	3.98
NEW	N	0.00026	0.00008	0.33	0.00024	0.94	0.00000	0.00	0.00147	5.74	0.00030	1.19	0.00137	5.37
MICA*070	N	0.00026	0.00008	0.33	0.00024	0.94	0.00000	0.00	0.00000	0.00	0.00000	0.00	0.00000	0.00
MICA*030	N	0.00018	0.00013	0.68	0.00000	0.00	0.00075	4.09	0.00760	41.22	0.00041	2.20	0.00027	1.49
MICA*052	N	0.00018	0.00038	2.05	0.00024	1.30	0.00000	0.00	0.00000	0.00	0.00314	17.03	0.00000	0.00
MICA*107N	Y	0.00016	0.00126	7.67	0.00000	0.00	0.00000	0.00	0.00000	0.00	0.00000	0.00	0.00000	0.00
MICA*141	Y	0.00014	0.00038	2.63	0.00000	0.00	0.00000	0.00	0.00000	0.00	0.00000	0.00	0.00027	1.92
MICA*089	Y	0.00012	0.00000	0.00	0.00000	0.00	0.00000	0.00	0.00000	0.00	0.00000	0.00	0.00000	0.00
MICA*119	Y	0.00010	0.00004	0.41	0.00000	0.00	0.00000	0.00	0.00000	0.00	0.00132	12.85	0.00000	0.00
MICA*136	Y	0.00009	0.00008	0.91	0.00000	0.00	0.00025	2.73	0.00000	0.00	0.00000	0.00	0.00055	5.96

*MICA* frequencies (protein/first-field resolution) were compared to the allele frequencies of the German population. A frequency ratio of more than twofold or less than half is highlighted in red or green, respectively. The cumulative frequency of all identified novel alleles in the respective population is indicated as ‘NEW’. Submitted alleles from this publication are marked with ‘Y’. Only the most frequent *MICA* alleles (based on the German population) are shown. See **Supplementary Data** for all alleles and a sortable table.

In the Polish population, allele frequencies were largely similar to the German population. The British/Irish population showed the highest MICA*008 frequency (51%) among all studied populations (**Table 2**; **Supplementary Data**).

Interestingly, the South African White population exhibited MICA allele frequencies closely resembling those of the other European populations. This contrasts with the South African Black population. Even though MICA*008 remained the most frequent *MICA* allele, its frequency was only 28% (43% in ZA_White). MICA*004 had a notably higher frequency (24%) in the South African Black population than in any other studied population, followed by MICA*002# (22%), MICA*019 (5% vs. 0.8% in ZA_White), and MICA*015 (5% vs. 0.3% in ZA_White). Conversely, other *MICA* alleles were underrepresented in the South African Black population: MICA*010# (0.05% vs. 8% in ZA_White), MICA*007 (0.2% vs. 4% in ZA_White), and MICA*017 (0.03% vs. 3% in ZA_White) (**Table 2**).

In the non-indigenous Chilean population, MICA*002# (31%) was the most frequent *MICA* allele, followed by MICA*008 (19%) and MICA*004 (10%). In the Turkish population residing in Germany, MICA*008 (21%) was followed by MICA*009# (20%) and MICA*002# (14%). Notably, MICA*016 had a frequency of 10% in this group, compared to only 2% in the German population.

Some novel alleles appeared to be population-specific. MICA*258 (n=26) and MICA*008:28 (n=45) were almost exclusively detected in individuals from South Africa that self-assigned as Black or Colored. Only one individual with MICA*008:28 self-assigned an Indian ethnic background. MICA*244 was exclusively identified in individuals of the ZA_White population (n=8) and all individuals with MICA*004:02 self-assigned a Polish or Russian ethnic background (n=6).

As expected, the characterization and submission of the novel alleles substantially reduced the cumulative frequency of novel alleles from 0.3% to 0.03% in the German population. However, less sequenced populations such as ZA_Black and DE_Turkey still reported higher cumulative novel allele frequencies (0.1%). This was likely due to the underrepresentation of these populations in the workflow and therefore lower prioritization for novel allele characterization.

Overall, in this independent cohort of 93,814 samples, we reidentified 120 of the 199 submitted novel MICA proteins. The remaining 79 were not detected again and are presumed to be rare.

### 3.4 Potential linkage of novel *MICA* alleles to *HLA-B*

It is well known that *MICA* is in strong linkage disequilibrium to *HLA-B* (23). However, due to the absence of phased genotype data, we are unable to determine *HLA-B* linkage information for every novel *MICA* allele. For alleles identified in multiple samples, though, we could infer the most likely linkage. For example, MICA*107N was identified 57 times in the cohort that was used for *MICA* frequency calculations, and all samples were positive for an HLA-B*14:02:01G allele, as well. Consequently, based on this co-occurrence, we conclude that MICA*107N and HLA-B*14:02:01G share a haplotype. Similarly, **Table 3** lists every novel *MICA* allele that was detected in at least 10 samples, of which all were reported with the given *HLA-B* allele.

**Table 3:** Linkage of novel *MICA* alleles to *HLA-B*.

*MICA* Allele	*HLA-B* linkage
MICA*089	HLA-B*35:01:01G
MICA*107N	HLA-B*14:02:01G
MICA*136	HLA-B*50:01:01G
MICA*141	HLA-B*49:01:01G
MICA*168	HLA-B*18:01:01G
MICA*185	HLA-B*47:01:01G
MICA*247	HLA-B*08:01:01G
MICA*258	HLA-B*13:03

## 4 Discussion

Recent research indicates potential future applications for *MICA* genotyping. On one hand, *MICA* informed donor selection has been associated with favorable outcomes in both HSC transplantation and solid organ transplantation (15–20). Similar to HLA, this would require *MICA* genotyping of patients and their (potential) donors. On the other hand, the regulatory pathways of the NKG2D receptor and its ligands have been proposed as promising targets for cancer immunotherapy (5). Innovative therapeutic approaches aim to increase the MICA/B density on the cell surface by enhancing MICA/B expression and/or inhibition of MICA/B shedding (41–43). For some of these potential future therapies, prior patient *MICA* genotyping might be necessary, e.g., to exclude variations in an antibody binding site.

A prerequisite for genotyping is an extensive and well maintained reference database, namely, the IPD-IMGT/HLA Database, which includes all HLA and related genes within the MHC complex (44). However, when we started *MICA* genotyping in 2017, the available data for *MICA* was still limited in comparison to the classical HLA genes (e.g., 107 described *MICA* alleles, 14% in full-length; release 3.35, January 2019). Consequently, we identified approximately ten times more novel *MICA* alleles (0.3%) than novel HLA alleles (0.02-0.04%) in the German population at that time (24). This not only complicates unambiguous reporting of genotyping results but also increases the workload during sequence data analysis.

After the characterization and submission of 603 *MICA* sequences to the IPD-IMGT/HLA Database, we were able to reduce the proportion of novel *MICA* alleles encountered during genotyping in samples from the German population to 0.03%. However, the fraction remains higher in South African Black and Turkish populations (0.1%) (**Table 2**). The reason for this is that we prioritized characterization of novel sequences according to their overall frequencies observed in our laboratory, which predominantly processes samples from Germany and Poland. Consequently, it can be assumed that additional, still-undescribed *MICA* alleles occur at higher frequencies in populations that were underrepresented in this study.

Among the characterized novel alleles are 199 distinct novel MICA proteins. Interestingly, all are similar to well-known MICA proteins, with unique amino acid variations randomly distributed across exons 2-5 (**Figure 3**). Due to the limitations of our high-throughput workflow that was used for variant identification, variations in exons 1 and 6 and small parts of exons 4 and 5 are severely underrepresented. Consequently, this limitation also applies to the current IPD-IMGT/HLA Database (release 3.60) where our novel alleles account for two thirds of all described alleles (**Figure 1B**).

In general, frequent MICA alleles have been functionally grouped by their mode of cell membrane attachment or by their binding affinity to NKG2D (8–12). Since most of our novel alleles harbor additional unique amino acid variations that have not been previously reported, we can only speculate that they may share functional characteristics with their closest known frequent alleles. It is worth noting that *MICB* seems to be as diverse as *MICA*, although only 307 *MICB* alleles are described in the current IPD-IMGT/HLA Database (release 3.60). In our study from 2020, we identified novel *MICB* alleles at a rate of 0.4% in the German population, but these have not yet been systematically characterized and submitted (24).

In this study, we confirmed *MICA* allele frequencies for the German population using an independent cohort of 48,618 samples (24). Additionally, we provide allele frequencies for 71 of our novel alleles (**Table 2**; **Supplementary Data**), with MICA*107N being the most frequent (0.02%). The *MICA* allele frequencies observed in other European populations (Polish and British/Irish), as well as the South African White population, were comparable to those found in the German population. In contrast, larger differences were observed in the South African Black, the non-indigenous Chilean and the Turkish population residing in Germany.

While some alleles are common across all populations (e.g., MICA*008, MICA*002#), others vary significantly. For example, MICA*010# has an allele frequency of 8% in the German population, 2% in the Turkish population with German residency and 0.05% in the South African Black population. Other studies reported 13% MICA*010 frequency in the Finnish population (45), and 17%-20% in South Korean and Chinese populations (46, 47). Another example is MICA*015, which has a frequency of 0.8% in the German population, 4.5% in the South African Black population, and was not detected in the Finnish or Asian studies (45–47). The novel allele MICA*258 was identified exclusively in the South African Black population, with a calculated allele frequency of 0.1%. Even though MICA-informed donor selection is not mandatory for HCT today and donor registries focus on the availability of an optimal HLA-matched donor for every patient, the characterization of such population-specific alleles presents an important step to population equity in donor registries (48). In conclusion, we report the identification, characterization and submission of 406 distinct novel *MICA* alleles and 197 sequence confirmations/extensions, along with *MICA* frequencies across several populations. These novel alleles have already been incorporated into the IPD-IMGT/HLA Database, thereby significantly broadening the reference for *MICA* genotyping.

## Data Availability

The datasets presented in this study can be found in online repositories. The names of the repository/repositories and accession number(s) can be found in the article/**Supplementary Material**.

## References

[1] Bahram S Bresnahan M Geraghty DE Spies T . A second lineage of mammalian major histocompatibility complex class I genes. Proc Natl Acad Sci. (1994) 91:6259–63. doi: 10.1073/pnas.91.14.6259, PMID: 8022771 PMC44180

[2] Bauer S Groh V Wu J Steinle A Phillips JH Lanier LL . Activation of NK cells and T cells by NKG2D, a receptor for stress-inducible MICA. Science. (1999) 285:727–9. doi: 10.1126/science.285.5428.727, PMID: 10426993

[3] Glienke J Sobanov Y Brostjan C Steffens C Nguyen C Lehrach H . The genomic organization of NKG2C, E, F, and D receptor genes in the human natural killer gene complex. Immunogenetics. (1998) 48:163–73. doi: 10.1007/s002510050420, PMID: 9683661

[4] Risti M Bicalho M da G . MICA and NKG2D: is there an impact on kidney transplant outcome? Front Immunol. (2017) 8:179. doi: 10.3389/fimmu.2017.00179, PMID: 28289413 PMC5326783

[5] Schmiedel D Mandelboim O . NKG2D ligands-critical targets for cancer immune escape and therapy. Front Immunol. (2018) 9:2040. doi: 10.3389/fimmu.2018.02040, PMID: 30254634 PMC6141707

[6] Zhao Y Chen N Yu Y Zhou L Niu C Liu Y . Prognostic value of MICA/B in cancers: a systematic review and meta-analysis. Oncotarget. (2017) 8:96384–95. doi: 10.18632/oncotarget.21466, PMID: 29221214 PMC5707108

[7] Li P Morris DL Willcox BE Steinle A Spies T Strong RK . Complex structure of the activating immunoreceptor NKG2D and its MHC class I–like ligand MICA. Nat Immunol. (2001) 2:443–51. doi: 10.1038/87757, PMID: 11323699

[8] Ashiru O López-Cobo S Fernández-Messina L Pontes-Quero S Pandolfi R Reyburn HT . A GPI anchor explains the unique biological features of the common NKG2D-ligand allele MICA*008. Biochem J. (2013) 454:295–302. doi: 10.1042/BJ20130194, PMID: 23772752

[9] Ashiru O Boutet P Fernández-Messina L Agüera-González S Skepper JN Valés-Gómez M . Natural killer cell cytotoxicity is suppressed by exposure to the human NKG2D ligand MICA*008 that is shed by tumor cells in exosomes. Cancer Res. (2010) 70:481–9. doi: 10.1158/0008-5472.CAN-09-1688, PMID: 20068167 PMC2817492

[10] Valés-Gómez M . The impact of glycosyl-phosphatidyl-inositol anchored MICA alleles on novel NKG2D-based therapies. Front Immunol. (2015) 6. doi: 10.3389/fimmu.2015.00193, PMID: 25964786 PMC4410604

[11] Steinle A Li P Morris DL Groh V Lanier LL Strong RK . Interactions of human NKG2D with its ligands MICA, MICB, and homologs of the mouse RAE-1 protein family. Immunogenetics. (2001) 53:279–87. doi: 10.1007/s002510100325, PMID: 11491531

[12] Luo Q Yin X Zhu Q Luo W Liu R Wei L . Two major human phenotypes of MICA molecules and their differential activation to NK cells via NKG2D receptor. Front Immunol. (2025) 16. doi: 10.3389/fimmu.2025.1563872, PMID: 40458406 PMC12127157

[13] Zuo J Mohammed F Moss P . The biological influence and clinical relevance of polymorphism within the NKG2D ligands. Front Immunol. (2018) 9:1820. doi: 10.3389/fimmu.2018.01820, PMID: 30166984 PMC6105697

[14] Isernhagen A Malzahn D Bickeböller H Dressel R . Impact of the MICA-129Met/val dimorphism on NKG2D-mediated biological functions and disease risks. Front Immunol. (2016) 7:588. doi: 10.3389/fimmu.2016.00588, PMID: 28018354 PMC5149524

[15] Isernhagen A Malzahn D Viktorova E Elsner L Monecke S von Bonin F . The MICA-129 dimorphism affects NKG2D signaling and outcome of hematopoietic stem cell transplantation. EMBO Mol Med. (2015) 7:1480–502. doi: 10.15252/emmm.201505246, PMID: 26483398 PMC4644379

[16] Parmar S del Lima M Zou Y Patah PA Liu P Cano P . Donor-recipient mismatches in MHC class I chain-related gene A in unrelated donor transplantation lead to increased incidence of acute graft-versus-host disease. Blood. (2009) 114:2884–7. doi: 10.1182/blood-2009-05-223172, PMID: 19654407 PMC4784289

[17] Fuerst D Neuchel C Niederwieser D Bunjes D Gramatzki M Wagner E . Matching for the MICA-129 polymorphism is beneficial in unrelated hematopoietic stem cell transplantation. Blood. (2016) 128:3169–76. doi: 10.1182/blood-2016-05-716357, PMID: 27811019

[18] Carapito R Jung N Untrau M Michel S Pichot A Giacometti G . Matching of MHC class I chain-related genes a and B is associated with reduced incidence of severe acute graft-versus-host disease after unrelated hematopoietic stem cell transplantation. Blood. (2014) 124:664–4. doi: 10.1182/blood.V124.21.664.664, PMID: 41496790

[19] Carapito R Jung N Kwemou M Untrau M Michel S Pichot A . Matching for the nonconventional MHC-I MICA gene significantly reduces the incidence of acute and chronic GVHD. Blood. (2016) 128:1979–86. doi: 10.1182/blood-2016-05-719070, PMID: 27549307 PMC5147017

[20] Carapito R Aouadi I Verniquet M Untrau M Pichot A Beaudrey T . The MHC class I MICA gene is a histocompatibility antigen in kidney transplantation. Nat Med. (2022) 28:989–98. doi: 10.1038/s41591-022-01725-2, PMID: 35288692 PMC9117142

[21] Jimenez Jimenez AM Spellman SR Politikos I McCurdy SR Devine SM Malki MMA . Allogeneic hematopoietic cell donor selection: contemporary guidelines from the NMDP/CIBMTR. Transplant Cell Ther. (2025) 31(12):973–88. doi: 10.1016/j.jtct.2025.07.004, PMID: 40619101 PMC12344661

[22] Sureda A Corbacioglu S Greco R Kröger N Carreras E . The EBMT Handbook: Hematopoietic Cell Transplantation and Cellular Therapies. Cham: Springer International Publishing (2024). 39437029

[23] Gao X Single RM Karacki P Marti D O’Brien SJ Carrington M . Diversity of MICA and linkage disequilibrium with HLA-B in two North American populations. Hum Immunol. (2006) 67:152–8. doi: 10.1016/j.humimm.2006.02.009, PMID: 16698437

[24] Klussmeier A Massalski C Putke K Schäfer G Sauter J Schefzyk D . High-throughput MICA/B genotyping of over two million samples: workflow and allele frequencies. Front Immunol. (2020) 11:314. doi: 10.3389/fimmu.2020.00314, PMID: 32153595 PMC7047279

[25] Lange V Böhme I Hofmann J Lang K Sauter J Schöne B . Cost-efficient high-throughput HLA typing by MiSeq amplicon sequencing. BMC Genomics. (2014) 15:63–3. doi: 10.1186/1471-2164-15-63, PMID: 24460756 PMC3909933

[26] Wagner I Schefzyk D Pruschke J Schöfl G Schöne B Gruber N . Allele-level KIR genotyping of more than a million samples: workflow, algorithm, and observations. Front Immunol. (2018) 9:2843. doi: 10.3389/fimmu.2018.02843, PMID: 30564239 PMC6288436

[27] Lang K Wagner I Schöne B Schöfl G Birkner K Hofmann JA . ABO allele-level frequency estimation based on population-scale genotyping by next generation sequencing. BMC Genomics. (2016) 17:374. doi: 10.1186/s12864-016-2687-1, PMID: 27207383 PMC4874024

[28] Sauter J Putke K Schefzyk D Pruschke J Solloch UV Bernas SN . HLA-E typing of more than 2.5 million potential hematopoietic stem cell donors: Methods and population-specific allele frequencies. Hum Immunol. (2021) 82:541–7. doi: 10.1016/j.humimm.2020.12.008, PMID: 33386168

[29] Schöfl G Lang K Quenzel P Böhme I Sauter J Hofmann JA . 2.7 million samples genotyped for HLA by next generation sequencing: lessons learned. BMC Genomics. (2017) 18:161. doi: 10.1186/s12864-017-3575-z, PMID: 28196473 PMC5309984

[30] Solloch UV Lang K Lange V Böhme I Schmidt AH Sauter J . Frequencies of gene variant CCR5-Δ32 in 87 countries based on next-generation sequencing of 1.3 million individuals sampled from 3 national DKMS donor centers. Hum Immunol. (2017) 78:710–7. doi: 10.1016/j.humimm.2017.10.001, PMID: 28987960

[31] Klussmeier A Putke K Klasberg S Kohler M Sauter J Schefzyk D . High population frequencies of MICA copy number variations originate from independent recombination events. Front Immunol. (2023) 14:1297589. doi: 10.3389/fimmu.2023.1297589, PMID: 38035108 PMC10684724

[32] Paech C Albrecht V Putke K Schöfl G Schöne B Schmidt AH . HLA-E diversity unfolded: Identification and characterization of 170 novel HLA-E alleles. HLA. (2021) 97:389–98. doi: 10.1111/tan.14195, PMID: 33527770 PMC8247977

[33] Albrecht V Zweiniger C Surendranath V Lang K Schöfl G Dahl A . Dual redundant sequencing strategy: Full-length gene characterisation of 1056 novel and confirmatory HLA alleles. HLA. (2017) 90:79–87. doi: 10.1111/tan.13057, PMID: 28547825 PMC6084308

[34] Putke K Albrecht V Paech C Pahlke M Schöne B Klasberg S . Full-length characterization of novel HLA-DRB1 alleles for reference database submission. Methods Mol Biol. (2024) 2809:145–56. doi: 10.1007/978-1-0716-3874-3_10, PMID: 38907896

[35] Albrecht V Pahlke M Sauter J Paech C Putke K Schmidt AH . Extensive analysis of genetic diversity in HLA-DMA, HLA-DMB, HLA-DOA and HLA-DOB: characterisation of 236 novel alleles. HLA. (2025) 105:e70231. doi: 10.1111/tan.70231, PMID: 40347049 PMC12063561

[36] Klasberg S Schmidt AH Lange V Schöfl G . DR2S: an integrated algorithm providing reference-grade haplotype sequences from heterozygous samples. BMC Bioinf. (2021) 22:236. doi: 10.1186/s12859-021-04153-0, PMID: 33971817 PMC8111713

[37] Schöne B Fuhrmann M Surendranath V Schmidt AH Lange V Schöfl G . TypeLoader2: Automated submission of novel HLA and killer-cell immunoglobulin-like receptor alleles in full length. HLA. (2019) 93:195–202. doi: 10.1111/tan.13508, PMID: 30821128 PMC6594033

[38] Schöne B Fuhrmann M Surendranath V Schmidt AH Lange V Schöfl G . Submitting novel full-length HLA, MIC, and KIR alleles with typeLoader2. Methods Mol Biol. (2024) 2809:157–69. doi: 10.1007/978-1-0716-3874-3_11, PMID: 38907897

[39] Paradis E Schliep K . ape 5.0: an environment for modern phylogenetics and evolutionary analyses in R. Bioinformatics. (2019) 35:526–8. doi: 10.1093/bioinformatics/bty633, PMID: 30016406

[40] Yu G . Using ggtree to visualize data on tree-like structures. Curr Protoc Bioinf. (2020) 69:e96. doi: 10.1002/cpbi.96, PMID: 32162851

[41] de Andrade LF Kumar S Luoma A Ito Y Alves da Silva PH Pan D . Inhibition of MICA and MICB Shedding Elicits NK cell–mediated Immunity against Tumors Resistant to Cytotoxic T cells. Cancer Immunol Res. (2020) 8:769–80. doi: 10.1158/2326-6066.CIR-19-0483, PMID: 32209637 PMC7269842

[42] Badrinath S Dellacherie MO Li A Zheng S Zhang X Sobral M . A vaccine targeting resistant tumours by dual T cell plus NK cell attack. Nature. (2022) 606:992–8. doi: 10.1038/s41586-022-04772-4, PMID: 35614223 PMC10253041

[43] Goulding J Yeh WI Hancock B Blum R Xu T Yang BH . A chimeric antigen receptor uniquely recognizing MICA/B stress proteins provides an effective approach to target solid tumors. Med. (2023) 4:457–477.e8. doi: 10.1016/j.medj.2023.04.004, PMID: 37172578 PMC10524375

[44] Barker DJ Maccari G Georgiou X Cooper MA Flicek P Robinson J . The IPD-IMGT/HLA database. Nucleic Acids Res. (2023) 51:D1053–60. doi: 10.1093/nar/gkac1011, PMID: 36350643 PMC9825470

[45] Koskela S Tammi S Clancy J Lucas JAM Turner TR Hyvärinen K . MICA and MICB allele assortment in Finland. HLA. (2023) 102:52–61. doi: 10.1111/tan.15023, PMID: 36919857

[46] Choi EJ Kim HJ Kim JH Baek IC . Distributions of MICA and MICB alleles typed by amplicon-based next-generation sequencing in South Koreans. HLA. (2024) 104:e15735. doi: 10.1111/tan.15735, PMID: 39470005

[47] Liu J Quan ZR Zhu TH Zhong YP Jiang RH Yang BN . Allele and haplotype frequencies of 17 HLA-related loci in Shenzhen Chinese population by next-generation sequencing. HLA. (2025) 105:e70148. doi: 10.1111/tan.70148, PMID: 40193066

[48] Sauter J Bernas SN Hlongwane X Mokomele P Mhlongo K Lange V . HLA haplotype frequency analysis reveals large patient benefits from stem cell donor recruitment in Black South African population. Blood Global Hematol. (2025) 1:100028. doi: 10.1016/j.bglo.2025.100028, PMID: 41737640

