# Supplementary figures and images for: Advances in MICA genotyping: characterization of 406 novel alleles and their frequencies in multiple populations
Source: Front Immunol. 2026 Mar 2;17:1741611. doi: 10.3389/fimmu.2026.1741611 (PMC12989365; doi:10.3389/fimmu.2026.1741611)

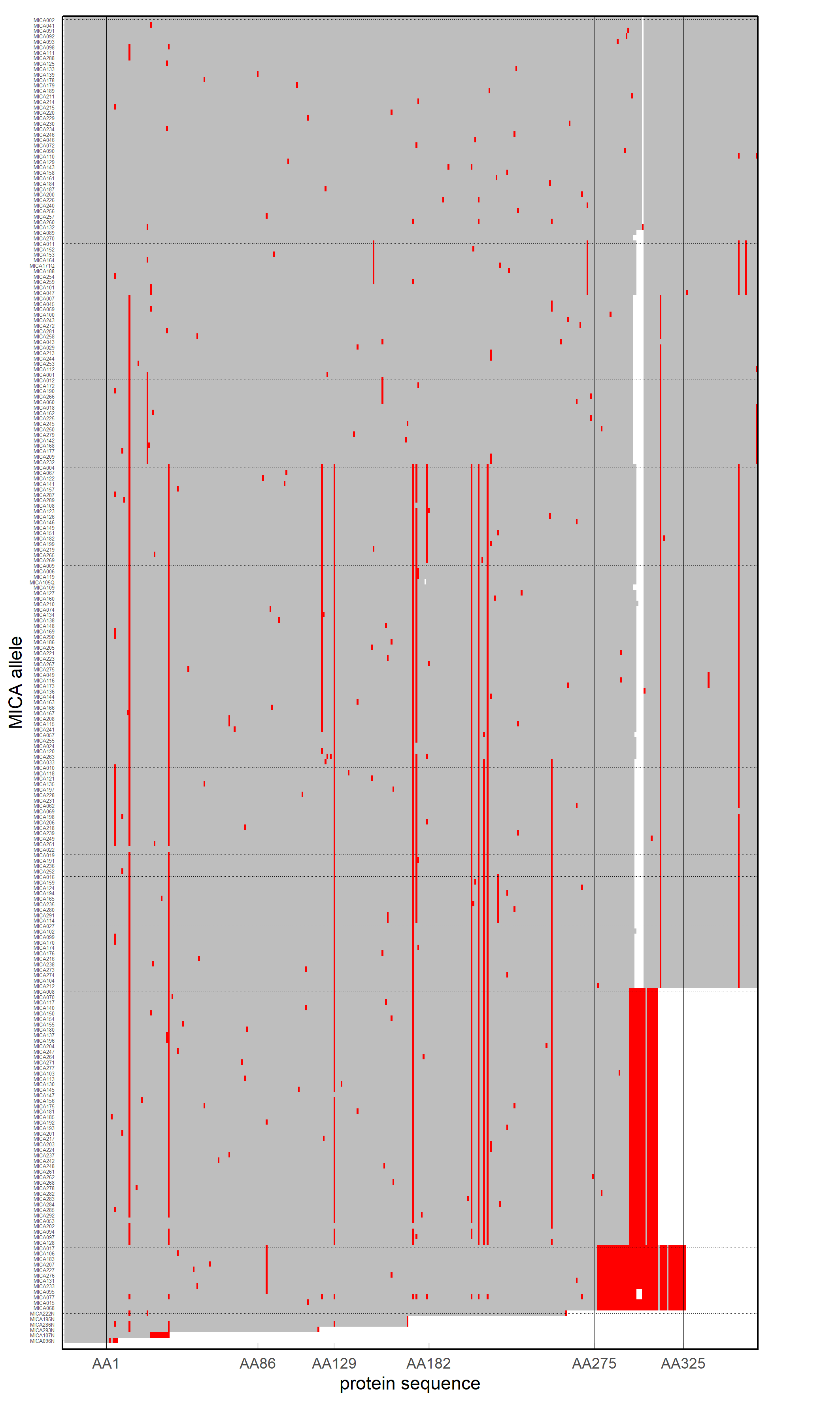

Supplement: Supplementary file 1 [file Image1.tif]
